# Pyrvinium selectively targets blast phase-chronic myeloid leukemia through inhibition of mitochondrial respiration

**DOI:** 10.18632/oncotarget.5615

**Published:** 2015-09-10

**Authors:** Wei Xiang, Jit Kong Cheong, Shi Hui Ang, Bryan Teo, Peng Xu, Kartini Asari, Wen Tian Sun, Hein Than, Ralph M. Bunte, David M. Virshup, Charles Chuah

**Affiliations:** ^1^ Department of Haematology, Singapore General Hospital, Singapore; ^2^ Program in Cancer and Stem Cell Biology, Duke-NUS Graduate Medical School, Singapore; ^3^ Office of Research, Duke-NUS Graduate Medical School, Singapore; ^4^ Department of Pediatrics, Duke University School of Medicine, Durham, NC, USA

**Keywords:** chronic myeloid leukemia, pyrvinium, mitochondrial respiration

## Abstract

The use of BCR-ABL1 tyrosine kinase inhibitors (TKI) has led to excellent clinical responses in patients with chronic phase chronic myeloid leukemia (CML). However these inhibitors have been less effective as single agents in the terminal blast phase (BP). We show that pyrvinium, a FDA-approved anthelminthic drug, selectively targets BP-CML CD34^+^ progenitor cells. Pyrvinium is effective in inducing apoptosis, inhibiting colony formation and self-renewal capacity of CD34^+^ cells from TKI-resistant BP-CML patients, while cord blood CD34^+^ are largely unaffected. The effects of pyrvinium are further enhanced upon combination with dasatinib, a second generation BCR-ABL1 TKI. In a CML xenograft model pyrvinium significantly inhibits tumor growth as a single agent, with complete inhibition in combination with dasatinib. While pyrvinium has been shown to inhibit the Wnt/β-catenin signalling pathway via activation of casein kinase 1α, we find its activity in CML is not dependent on this pathway. Instead, we show that pyrvinium localizes to mitochondria and induces apoptosis by inhibiting mitochondrial respiration. Our study suggests that pyrvinium is a useful addition to the treatment armamentarium for BP-CML and that targeting mitochondrial respiration may be a potential therapeutic strategy in aggressive leukemia.

## INTRODUCTION

Chronic myeloid leukemia (CML) is a malignant hematopoietic stem cell disorder caused by the oncogenic BCR-ABL1 fusion protein. Although the introduction of BCR-ABL1 tyrosine kinase inhibitors (TKI) has improved clinical responses and outcomes significantly, blast phase (BP) disease remains a major therapeutic challenge [1]. The molecular mechanisms leading to BP disease progression are complex and involve inhibition of tumor suppressors, genomic instability and block of myeloid differentiation [2]. Novel strategies are currently being investigated in early phase clinical trials or preclinical studies. These include activation of the tumor suppressor, protein phosphatase 2A or inhibition of leukemia stem cell self-renewal by targeting the Wnt/β-catenin pathway through inhibition of MAP kinase interacting serine/threonine kinase (MNK)-eukaryotic translation initiation factor 4E (eIF4E) axis [3, 4].

Targeting cancer metabolism, such as oxidative phosphorylation, has recently become an attractive therapeutic strategy in human leukemia [5]. In contrast to normal progenitor cells, acute myeloid leukemia (AML) progenitor cells have increased mitochondrial biogenesis and are more dependent on mitochondrial respiration rather than glycolysis for energy production to maintain survival [6, 7]. Inhibition of oxidative phosphorylation or mitochondrial translation selectively kills AML progenitor cells but not normal bone marrow or peripheral blood progenitor cells, suggesting that the unique dependence of leukemia primary cells on oxidative phosphorylation can be exploited therapeutically [6, 7].

Pyrvinium is a FDA-approved anthelminthic drug used for the treatment of pinworm infections [8]. Recent studies have shown that pyrvinium selectively inhibits the growth of tumor cells of diverse tissue origins, including pancreas, colon, breast, brain, myeloma and erythroleukemia [9–17]. It has also been shown that pyrvinium exhibits increased cytotoxicity in tumor cells under hypoglycemic conditions and enhances the effects of conventional chemotherapeutic drugs in tumor xenografts [11, 13, 18]. The mechanism of action of pyrvinium appears to vary in different tumor types. Pyrvinium has been reported to inhibit the growth of colon cancer cells through allosteric activation of casein kinase 1α (CK1α) and destabilization of β-catenin [15]. Pyrvinium also inhibits medulloblastoma through attenuating hedgehog signalling in a CK1α dependent manner [16]. However, pyrvinium has also been shown to inhibit the proliferation of myeloma and erythroleukemia cells by suppressing the mitochondrial respiratory complex I [12]. Other mechanisms that have been implicated include targeting unfolded protein response and autophagy [11, 13].

In this study, we investigated the effect of pyrvinium and its combination with the BCR-ABL1 TKI, dasatinib in BP-CML. We found that pyrvinium selectively induces apoptosis, inhibits colony formation and self-renewal of CD34^+^ progenitor cells from TKI-resistant BP-CML patients. We also show that pyrvinium enhances the effect of dasatinib *in vitro* and *in vivo*. Finally, we demonstrate that the anti-tumor effects of pyrvinium in BP-CML are independent of CK1α inhibition and are largely attributed to its inhibition of mitochondrial respiration.

## RESULTS

### Pyrvinium is active against CML cell lines and synergistic in combination with dasatinib *in vitro* and *in vivo*

**Figure 1:**
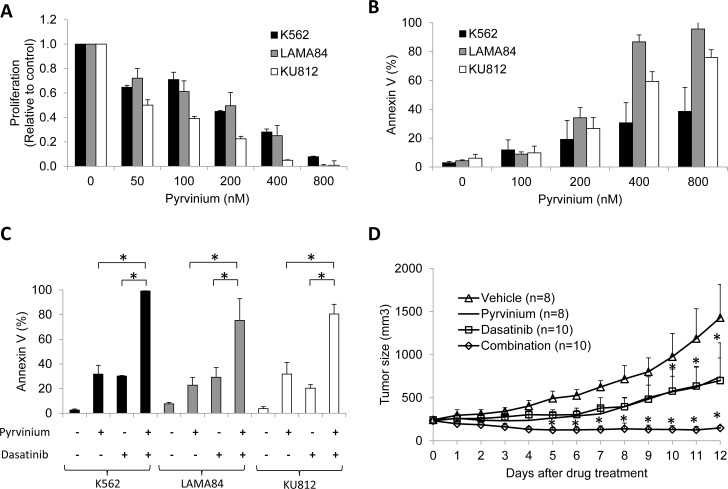
Pyrvinium alone, and in combination with dasatinib, inhibits growth of CML cells *in vitro* and *in vivo* **a.** Pyrvinium significant decreases proliferation of K562, LAMA84 and KU812 cells in a dose-dependent manner. Results shown are the fold change relative to control. **b.** Pyrvinium significantly induces apoptosis of BP-CML cells. **c.** Combination of pyrvinium and dasatinib is more superior in apoptosis induction than single drug treatment. The concentrations of pyrvinium and dasatinib used in combination studies are 400, 150, 200 nM and 0.8, 0.2, 0.2 nM in K562, LAMA84 and KU812 cells respectively. These data are derived from three independent experiments. **d.** Combination of pyrvinium and dasatinib arrests growth of CML xenografts. SCID mice bearing K562 tumor xenografts at the flanks were treated with equal volume of vehicles (citric acid and DMSO/saline, *n* = 8), 1 mg/kg dasatinib (*n* = 8) by oral gavage, 0.5 mg/kg pyrvinium (*n* = 10) by intraperitoneal injection or both drugs (*n* = 10). *p < 0.01, compared to untreated controls or single arm treatment.

We investigated the effects of pyrvinium alone and in combination with dasatinib on the proliferation and apoptosis in human CML cell lines (K562, LAMA84 and KU812). Exposure to pyrvinium alone inhibited proliferation of the CML cells in a dose-dependent manner, with IC_50_ of 50-200 nM (Figure 1a). Pyrvinium also induced apoptosis in these cells, particularly LAMA84 and KU812, as assessed by flow cytometry for Annexin V staining (Figure 1b). To determine the combination effects of pyrvinium and dasatinib, we designed combination studies based on the methods proposed by Chou and Talalay [19] Combination indices (CI) at 50%, 75% and 90% growth inhibition are summarised in Table 1. Synergy, as defined by a CI < 1 between pyrvinium and dasatinib, was observed at all effect levels. The combination of pyrvinium and dasatinib was also superior in inducing apoptosis in the CML cell lines (Figure 1c).

**Table 1:** Combination of pyrvinium and dasatinib is synergistic in inhibiting proliferation of cultured BP-CML cells

	CI at IC_50_	CI at IC_75_	CI at IC_90_
K562	0.61 ± 0.03	0.51±0.08	0.47±0.12
LAMA84	0.70 ± 0.08	0.55±0.05	0.47±0.05
KU812	0.76±0.20	0.63±0.06	0.60±0.03

The IC_50_ values of pyrvinium and dasatinib were obtained from relative proliferation versus log [drug] graphs plotted using MTS data with Prism 5. The approximate ratios of the concentrations of pyrvinium and dasatinib for combination studies were determined from the average IC_50_ values of each drug in the single arm experiments. Combination index (CI) was calculated using the Calcusyn software. CI of less than 1 indicates synergism; CI equals to 1 indicates additivity; and CI of greater than 1 indicates antagonism of the two drugs in combination.

We next evaluated the effects of pyrvinium *in vivo* and tested whether combination with dasatinib resulted in greater efficacy than with single drug. Using an established CML xenograft mouse model [20], we injected K562 cells subcutaneously into the flank of SCID mice. Once tumors reached approximately 200mm^3^, the mice were treated with intraperitoneal pyrvinium 0.5 mg/kg daily, oral dasatinib 1 mg/kg daily or a combination of both. The mice in all 3 groups tolerated the treatment well, as assessed by body weight (Supplemental Figure S1). Pyrvinium delayed tumor growth beginning at 4 days of the initial treatment and its inhibitory effect was observed throughout the duration of treatment (Figure 1d). Of note, the inhibitory effect of pyrvinium 0.5 mg/kg was similar to dasatinib 1 mg/kg. When both drugs were combined, tumor growth was completely inhibited.

### Pyrvinium selectively targets BP-CML CD34^+^ progenitor cells and acts synergistically with dasatinib

**Figure 2:**
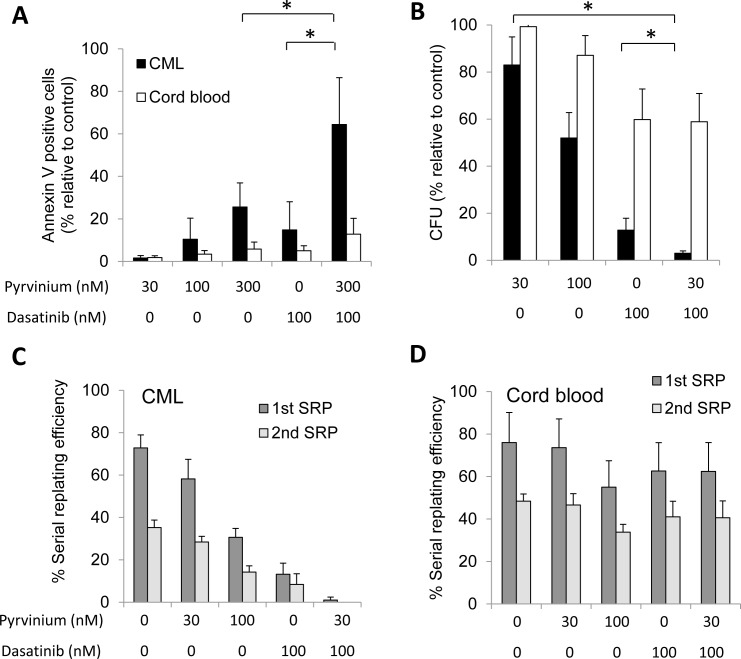
Pyrvinium alone and in combination with dasatinib selectively arrests growth of patient-derived BP-CML CD34^+^ progenitor cells *ex vivo* **a.** Pyrvinium selectively induces apoptosis of BP-CML, but not CB, CD34^+^ cells and combination of pyrvinium and dasatinib is superior in inducing apoptosis than single drug alone. Results shown are the average percentage of induced apoptosis above the respective controls. Pyrvinium significantly reduces colony formation **b.** and serial replating capacity **c.** and **d.** of CML, but not CB, CD34^+^ cells. Results shown are the percentage of the number of positive wells relative to the total number of colonies replated for the serial replating assays. Graphs presented are mean of the results obtained from patient-derived BP-CML or CB samples (CML, *n* = 5; CB, *n* = 5). Error bars represent standard deviation. *p < 0.01, compared to untreated controls or single arm treatment.

An important feature of targeted therapy is the ability to be selective in retaining activity against leukemia cells while sparing normal cells. Compared to chronic phase CML, TKI inhibitors are less effective as single agents in BP-CML cells. We therefore examined the effects of pyrvinium, dasatinib or the combination on CD34^+^ cells isolated from BP-CML patients or from cord blood (patient clinical information is in Supplemental Table 1). Consistent with our CML cell line results, pyrvinium induced dose-dependent apoptosis in CD34^+^ cells in BP-CML patients. The combination of pyrvinium and dasatinib further enhanced apoptosis compared to single agent therapy. Importantly, we did not observe enhanced apoptosis in drug combination-treated cord blood CD34^+^ cells (Figure 2a and Supplemental Table 2), indicating that pyrvinium and its combination with dasatinib exhibit selective toxicity against BP-CML *vs*. cord blood CD34^+^ progenitor cells. In addition, the combination of pyrvinium and other TKIs, such as imatinib and nilotinib, also induced significantly more apoptosis of BP-CML CD34 cells than single drug alone (Supplemental Figure S2).

The propensity to self-renew, proliferate and differentiate are hallmark features of stem/progenitor cells [21]. To test whether pyrvinium affects proliferation and self-renewal of BP-CML CD34^+^ cells, we performed colony-forming and serial replating assays. We found that pyrvinium decreased colony formation and self-renewal capacity of BP-CML CD34^+^ cells in a dose-dependent manner (Figures 2b–2c). We noted that cord blood CD34^+^ cells were less sensitive to increasing doses of pyrvinium exposure. In addition, colony formation and self-renewal of BP-CML but not cord blood CD34^+^ cells were completely abolished when they were treated with a combination of dasatinib and pyrvinium (Figures 2b–2c and Supplemental Tables 3-4). Hence, pyrvinium alone and its combination with dasatinib preferentially target BP-CML compared to cord blood CD34^+^ progenitors by inhibiting their proliferation and self-renewal capacity.

### Pyrvinium acts on CML in a CK1α-independent manner

The direct anti-cancer molecular targets of pyrvinium have rarely been elucidated [12, 13, 15]. Thorne *et al*. demonstrated that the anti-colon cancer effects of pyrvinium were due to its allosteric activation of CK1α and subsequent suppression of Wnt/β-catenin signalling [15]. Given that Wnt/β-catenin signalling has been shown to be activated in BP-CML [22], we tested whether CK1α or destabilization of β-catenin is required for the anti-CML effects of pyrvinium.

**Figure 3:**
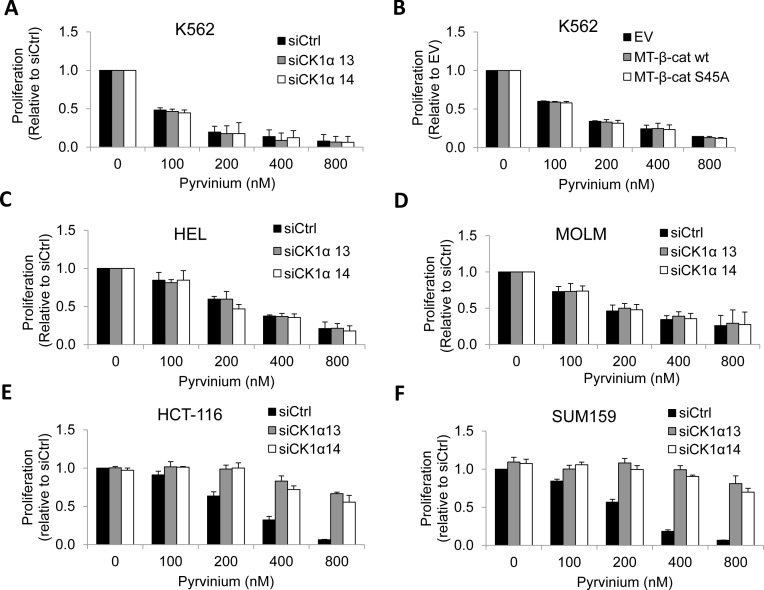
Reliance on CK1α for pyrvinium-induced cancer cell arrest is context-dependent **a.** CK1α-depleted K562 CML cells remain sensitive to pyrvinium. Cells are electroporated with 100 nM siCtrl or siCK1α (#13 or #14) and cultured for 24 hours prior to MTS assays. **b.** Overexpression of wild-type or stabilized β-catenin (S45A) in K562 cells fails to rescue the growth inhibitory effects of pyrvinium. Cells are electroporated with 1.5 μg pCS2-MT (EV), pCS2-MT-β-cat wt or pCS2-MT-β-cat S45A and cultured for 24 hours prior to MTS assays. CK1α-depleted AML cells HEL **c.** and MOLM **d.** remain sensitive to pyrvinium. (**e** and **f**) Depeletion of CK1α abolishes prvinium-induced growth inhibition of HCT-116 colon cancer and SUM159 breast cancer cells. siCtrl- or siCK1α-expressing cells are treated with DMSO or pyrvinium (100, 200, 400 and 800 nM).

We first depleted CK1α in K562 cells with two independent CK1α siRNA but observed that these cells remained sensitive to pyrvinium (Figure 3a and Supplemental Figure S3a). Similarly, overexpression of wild-type or stabilized β-catenin (S45A) in K562 cells failed to rescue the growth inhibitory effects of pyrvinium (Figure 3b and Supplemental Figure S3b). We found that depletion of CK1α in two other AML cell lines (HEL and MOLM) also failed to reverse the growth inhibitory effects of pyrvinium (Figures 3c–3d and Supplemental Figure S3c). The data indicates that pyrvinium acts on leukemia cells in a CK1α and β-catenin-independent manner. Since pyrvinium inhibits proliferation of HCT-116 through activating CK1α [15], we next determined whether the target of pyrvinium is cell-type specific Consistent with the findings of Throne et al., pyrvinium-induced growth inhibition of HCT-116 colon cancer and SUM159 breast cancer cells was abolished by depletion of CK1α (Figures 3e–3f and Supplemental Figure S3c), suggesting that CK1α is required for the mechanism of action of pyrvinium in breast and colon cancer. Taken together, our data suggest that pyrvinium acts in a cancer cell-type specific manner and additional important molecular targets of pyrvinium are present in CML.

### Pyrvinium preferentially localizes in mitochondria of CML cells, rapidly inhibits mitochondrial respiratory capacity and decreases ATP levels

**Figure 4:**
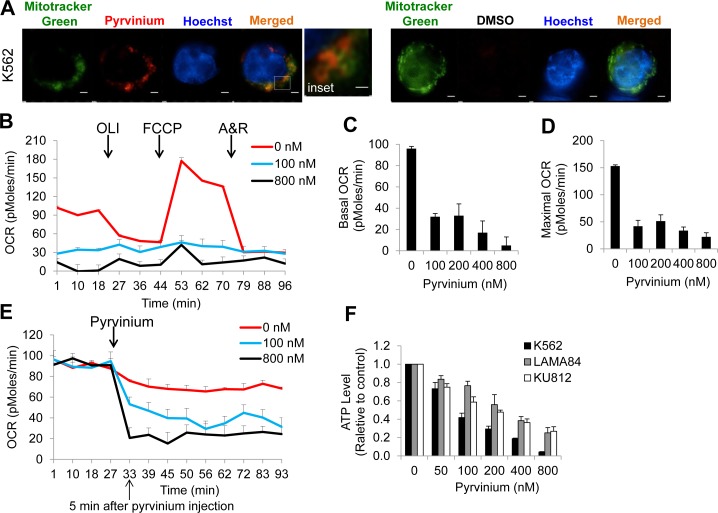
Pyrvinium localizes to the mitochondrial and blocks oxidative phosphorylation of CML cells **a.** Pyrvinium preferentially localizes to the mitochondria of CML cells. Super resolution microscopy of live K562 cells co-incubated with pyrvinium (red) and the mitochondrial marker, MitoTracker Green. Hoechst 33342 stains the nuclei. Scale bar is 5μm. **b.-d.** Pyrvinium significantly decreases basal and maximal OCR (oxygen consumption rate) in CML cells. K562 cells were treated with DMSO or pyrvinium for 24 hours and OCR was measured without (the first three measurements) and in the presence of (measurements 4-12) oligomycin (OLI, 1 μg/ml), FCCP (0.4 μM) and Antimycin A and Rotenone combination (A&R, 2.5 μM and 2.5 μM). OLI, FCCP and A&R were injected into the wells at the time indicated by arrows. Basal OCR is calculated as the mean of measurements of the first three. Maximal OCR is calculated as the mean of the measurement 7 - 9. **e.** Pyrvinium rapidly reduces oxidative phosphorylation of BP-CML cells. OCR was measured without (the first four measurements) and with different concentrations of pyrvinium (measurements 5-13) every 5 minutes. Pyrvinium was injected into the wells at the time indicated by arrow. **f.** Pyrvinium decreases ATP levels of multiple CML cell lines.

Given that pyrvinium inhibits proliferation of myeloma/erythroleukemia cells by suppressing mitochondrial respiration [12], we next investigated whether the target of pyrvinium in CML cells exists in the mitochondria. Pyrvinium is a quinolone-derived cyanine dye that fluoresces red. We assessed its subcellular location in CML cells via fluorescent confocal microscopy and the mitochondria marker, Mitotracker Green. We found that pyrvinium stains K562, LAMA84 and KU812 cells within 5 minutes (Figure 4a and Supplemental Figure S4a). Importantly, pyrvinium co-localizes with Mito-tracker Green, suggesting that the target of pyrvinium resides in the mitochondria of CML cells.

Next, we tested whether pyrvinium affects physiologic functions of the mitochondria of CML cells. We found that CML cells that were treated with pyrvinium had a significantly reduced baseline oxygen consumption rate (OCR) and were non-responsive to uncoupling of mitochondrial oxidative phosphorylation via FCCP (Figures 4b–4d; Supplemental Figures S4b-S4g). Consistent with the mitochondrial localization data, the pyrvinium-induced decrease in oxidative phosphorylation (measured by OCR) was achieved within 5 minutes of drug exposure (Figure 4e; Supplemental Figure S5). Furthermore, a significant decline in cellular ATP levels was observed in pyrvinium-treated CML cells as compared to their vehicle-treated counterparts (Figure 4f). Taken together these data show that pyrvinium significantly and directly inhibits basal and spare respiratory capacity of CML cells to induce an acute energy crisis.

### Mitochondrial respiratory chain-deficient CML ρ^0^ cells are insensitive to pyrvinium

**Figure 5:**
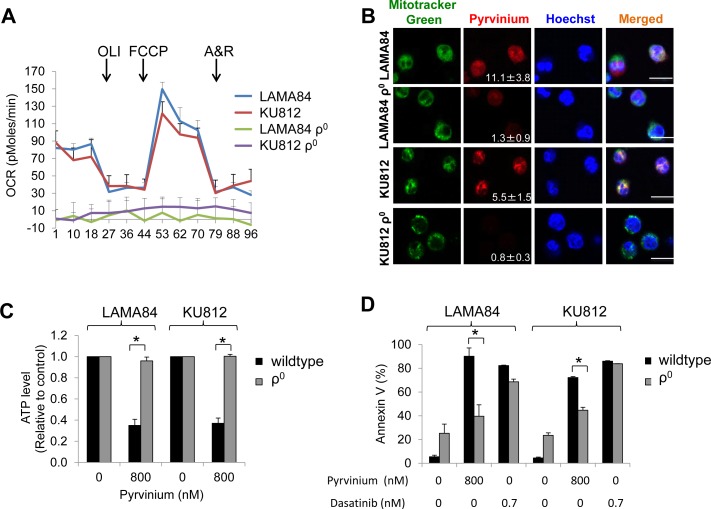
Mitochondrial respiratory chain-deficient CML ρ^0^ cells are insensitive to pyrvinium **a.** LAMA84 ρ^0^ and KU812 ρ^0^ cells have minimal basal as well as spare oxygen consumption. OCR was measured using untreated cells without and in the presence of oligomycin (OLI, 1 μg/ml), FCCP (0.4 μM) and Antimycin A and Rotenone combination (A&R, 2.5 μM and 2.5 μM). **b.** Mitochondria localization of pyrvinium is significantly reduced in CML ρ^0^ cells. Confocal microscopy of live CML cells co-incubated with pyrvinium (red; numbers represent mean pixel intensity of red signal from four different 63X magnification fields ± standard deviation) and the mitochondrial marker, MitoTracker Green. Hoechst 33342 stains the nuclei. Scale bar is 25 μm. **c.** CML ρ^0^ cells are resistant to pyrvinium-induced depletion of ATP. **d.** Pyrvinium but not dasatinib is ineffective in inducing apoptosis in CML ρ^0^ cells. *p < 0.01, compared to CML cells.

To investigate whether pyrvinium targets a mitochondrial genome-encoded component of oxidative phosphorylation in CML, we generated CML ρ^0^ cells that lack mitochondrial DNA and are incapable of performing mitochondrial respiration [23]. We confirmed that gene expression from mitochondrial DNA (*MT-ND6* and *MT-CO2*), but not nuclear DNA (*SDHA*), was significantly lower in the ρ^0^ cells derived from LAMA84 and KU812, as compared to their parental lines (Supplemental Figure S6). These ρ^0^ cells also exhibit a significantly reduced baseline OCR (Figure 5a), indicative of defective mitochondrial respiration. Notably, the localization of pyrvinium to the mitochondria of LAMA84 and KU812 ρ^0^ cells was markedly reduced (Figure 5b). Furthermore, the ATP levels of LAMA84 and KU812 ρ^0^ cells remained constant even in the presence of pyrvinium exposure (Figure 5c). These ρ^0^ cells were also resistant to apoptosis induction by pyrvinium but not dasatinib (Figure 5d). The inhibitory effect of pyrvinium on the proliferation on CML ρ^0^ cells was not determined due to inadequate proliferation of ρ^0^ cells (Supplemental Figure S6B and S6C). Collectively, our data indicate that pyrvinium specifically targets mitochondrial respiration, most likely by binding to and inhibiting the function of a component of oxidative phosphorylation encoded by the mtDNA.

## DISCUSSION

The advent of BCR-ABL TKIs in the past 15 years has greatly improved the prognosis of CML. Although these TKIs curb the unchecked growth of CML progenitors and their progeny, they fail to eliminate leukemia stem cells (LSC) that may be the ultimate driver of disease relapse [24]. Targeting metabolic pathways for cancer therapy has attracted attention ever since Warburg's seminal discovery of aerobic glycolysis [25]. However, recent studies have suggested that Warburg's paradigm of reprogramming energy metabolism may not necessarily apply to cancer stem cells. Consistent with the emerging evidence showing that LSC rely heavily on oxidative phosphorylation for survival [6, 7], we herein report the identification of an FDA-approved drug, pyrvinium, that selectively eliminates primary BP-CML CD34^+^ progenitor cells through induction of mitochondrial respiration blockade. Importantly, pyrvinium induces apoptosis, inhibits colony formation and blocks self-renewal of CD34^+^ cells isolated from BP-CML patients who harbour TKI-resistant BCR-ABL1 kinase mutations (Supplemental Table 1). The combination of pyrvinium and dasatinib is superior to either drug alone (Figure 2), indicating that the drug combination works synergistically to eradicate cultured CML cells and CML xenografts. Furthermore, pyrvinium alone or in combination with dasatinib showed no overt toxicity to CD34^+^ cells from normal cord blood (Figure 2). Taken together, the exquisite reliance of of BP-CML stem/progenitor cells on oxidative phosphorylation may offer a unique therapeutic opportunity in patients with TKI-resistant disease. Our ongoing studies will determine whether the combination of pyrvinium with dasatinib or other TKIs is also effective in other preclinical CML blast crisis mouse models [26].

The molecular target of pyrvinium has been hotly debated over the past few years. This is largely attributed to the difference in experimental readouts used to test this FDA-approved anthelminthic compound for its potential utility in cancer research and treatment. While reports have clearly demonstrated that pyrvinium allosterically activates CK1α to block Wnt/β-catenin signalling in colon cancer and hedgehog signalling in medulloblastomas respectively [15–17], others have shown that CK1α is not the *bona-fide* target of pyrvinium in transformed cells of the embryonic kidney (HEK293T) and leukemia origin [12, 13, 27]. Notably, Harada *et al* showed that the target of pyrvinium in myeloma/erythroleukemia cells is the mitochondrial respiratory complex I [12]. Putting all these findings into a unifying perspective, we showed that the relative dependence of pyrvinium on CK1α for its anti-proliferative property differs greatly between breast and colon cancer cells and leukemia cells. While we and others have shown that the presence of CK1α is absolutely required for pyrvinium to exert its anti-cancer effects on HCT-116 colon carcinoma cells [17], we also demonstrated that CK1α depletion is insufficient to rescue the growth of CML and AML cell lines exposed to pyrvinium (Figure 3a–3d and Supplemental Figure S3). Consistent with the work of Harada et al, we showed that pyrvinium-induced cell death was significantly reduced in CML ρ^0^ cells that cannot perform mitochondrial respiration (Figure 5). These data indicate that pyrvinium differentially targets solid and blood cancers via CK1α activation and mitochondrial respiration inhibition, respectively. Given that the localization of pyrvinium to the mitochondria is markedly reduced in CML ρ^0^ cells (Figure 5b), we speculate that pyrvinium likely targets a component of the electron transport chain encoded by the mitochondrial DNA. The mammalian mitochondrial DNA encodes 13 polypeptides including subunits for mitochondrial respiratory complex I, III and IV [23]. Since pyrvinium inhibits mitochondrial respiratory complex I activities in myeloma cells [12], we speculate that pyrvinium may target mitochondrial DNA encoded subunits of complex I in CML cells.

Recent studies indicate that some tumors are highly dependent on mitochondrial respiration for survival [28, 29]. Targeting mitochondrial respiration by either mitochondrial translation inhibition or BCL-2 inhibition selectively eradicates AML stem/progenitor cells due to their higher dependence on mitochondrial respiration than normal hematopoietic counterparts [6, 7]. Based on our findings that pyrvinium inhibits mitochondrial respiration and targets BP-CML cells more effectively than normal hematopoietic cells, we hypothesize that CML stem/progenitor cells are more metabolically active and dependent on mitochondrial respiration than are normal hematopoietic counterparts. As our study demonstrates, this dependency can be exploited by rationally targeted therapy.

In conclusion, we demonstrate that pyrvinium selectively targets BP-CML cells through inhibition of mitochondrial respiration. Blockade of mitochondrial respiration, combined with clinically approved BCR-ABL1 TKIs, induces BP-CML cell death *in vitro* and *in vivo*. Hence, targeting mitochondrial respiration may represent a new therapeutic strategy against aggressive leukemia.

## MATERIALS AND METHODS

### Primary cells

CD34^+^ cells were purified from bone marrow or peripheral blood mononuclear cells of BP-CML patients or from cord blood cells using CD34 MicroBead kit (Miltenyi Biotec, Germany). CD34^+^ cells were cultured in serum free medium using StemPro complete medium (Life Technologies, CA, US) supplemented with cytokines (stem cell factor, 200 pg/mL; granulocyte-macrophage colony-stimulating factor, 200 pg/mL; macrophage inflammatory protein-1α, 200 pg/mL; granulocyte colony−stimulating factor, 1000 pg/mL; leukemia inhibitory factor, 50 pg/mL; and interleukin 6, 1000 pg/mL) similar to that found in stroma-conditioned medium from long-term bone marrow [30]. Primary CML samples were obtained from patients seen at the Singapore General Hospital. Written informed consent was obtained from all patients under institutional review board-approved protocols. Cord blood samples were obtained from the Singapore Cord Blood Bank.

### Cell lines

Human CML cell lines, K562, LAMA84 (kind gift from Dr. Junia V. Melo), and KU812; and acute myeloid leukemia (AML) cell lines, HEL and MOLM (kind gift from Dr. S. Tiong Ong) were grown in RPMI1640 medium containing 4 mM L-glutamine (Life Technologies, CA, US) and 10% fetal bovine serum (FBS) (Hyclone, UK). Human HCT-116 colon cancer and SUM159 breast cancer cell lines were purchased from American Type Culture Collection (ATCC) and cultured in accordance to ATCC's instruction. Mitochondria DNA-deficient LAMA84 ρ^0^ and KU812 ρ^0^ cells were established by growing in RPMI1640 medium containing 10% FBS, 2 μg/ml ethidium bromide (EtBr), 4 mM L-glutamine, 50 μg/ml uridine, 100 μg/ml sodium pyruvate (Sigma, MO, US) for 50 days, and thereafter maintained in above media without EtBr [31].

### Drugs

Pyrvinium (P0027, Sigma, MO, US) and dasatinib (LC laboratories, LA, US) were dissolved in DMSO. For the animal experiments, pyrvinium was dissolved in 50%/50% DMSO/saline and dasatinib was dissolved in 80 mM citric acid (pH 3.1).

### RNAi of human CK1α expression

Exponentially-growing leukemia cells at 1 × 10^6^ were electroporated with 100 nM non-targeting siRNA (siCtrl; D-001810-0X), human CK1α-specific siRNAs or wildtype/stabilized β-catenin using the Amaxa Nucleofector^TM^ kit (Lonza,Germany), for 24h prior to the indicated drug treatment. 5 × 10^4^ HCT-116 or SUM159 cells were plated in 12-well plates and transfected with 100 nM non-targeting siRNA (siCtrl; D-001810-0X) or human CK1α-specific siRNAs using Dharmafect Transfection Reagent (Dharmacon RNAi Technologies), in accordance with the manufacturer's instructions, for 24h prior to the indicated drug treatment. The target sequences of human CK1α-specific ONTARGETplus siRNAs (Dharmacon RNAi Technologies) are siCK1α 13 (J-003957-13): GCGAUGUACUUAAACUAUU, siCK1α14 (J-003957-14): GGAAUCAUUAGGAUAUGUU.

### Measurement of apoptosis

Assessment of apoptosis was performed by Annexin V staining according to the manufacturer's instructions (Beckman Coulter, France). After 72 hours of drug incubation, cells were stained with Annexin V-FITC and analysed on a Beckman Coulter FC500.

### Cell proliferation assay

CML cell lines were plated on 96-well-plates and treated with graded doses of each drug for 72 hours. Cell proliferation activity was evaluated by the MTS proliferation assay kit (Life technologies, CA, US). Combination studies were designed based on the methods proposed by Chou and Talalay [19]. Briefly, the concentrations of pyrvinium and dasatinib required to inhibit 50% proliferation (IC_50_) was determined in the single arm experiments. The cells were then treated with increasing doses of pyrvinium or dasatinib or an equipotent constant-ratio combination of both drugs. The combination index (CI) at 50%, 75% and 90% growth inhibition was calculated using the Calcusyn software (Biosoft, UK) to determine if the combination was synergistic (CI<0.9), additive (CI 0.9-1.1) or antagonistic (CI>1.1). The use of crystal violet staining for cell growth assays has been previously described [32].

### Colony-forming and serial replating assays

Primary CD34^+^ cells (1000-5000) together with drugs were plated in HSC-CFU complete methylcellulose medium (Miltenyi Biotec, Germany). After 2 weeks, the number of colonies was scored and clusters with more than 100 cells were counted as a colony. For serial replating assays, individual colonies formed in CFU assays were picked and replated in HSC-CFU complete methylcellulose in a 96-well format. After 2-week incubation, wells were scored as positive or negative for the presence of colonies. Further rounds of serial replating were performed until no more colonies formed. Serial replating capacity is determined by the percentage of final number of positive wells among total number of colonies plated.

### Mitochondria labelling and pyrvinium localization

Cell lines were incubated with either DMSO or 200 nM pyrvinium for 5 minutes in a cell culture incubator. Cells were then washed with plain RPMI1640 and incubated with 100 nM Mitotracker Green (Invitrogen, CA, US) for 30 minutes in the cell culture incubator. Hoechst stain (Invitrogen, CA, US) was added to the cells and incubated for an additional 15 minutes. Cells were washed with plain RPMI1640 for three times and pelleted via centrifugation at 1200 rpm for 5 minutes. Cell pellets were resuspended with 200 μl plain RPMI1640 and transferred to NUNC^TM^ Lab-Tek^TM^ II chamber slide systems (Thermo Scientific, NY, US) for live cell imaging. Images were acquired with 3D-Structure Illumination Microscopy (3D-SIM) using a super resolution microscope (Nikon, Japan) equipped with an iXonEM+885 EMCCD camera (Andor) mounted on a Nikon Eclipse Ti-E inverted microscope with a CFI Apo TIRF (1006/1.40 oil) objective and processed with the NIS-Elements AR software.

### Metabolic assays

Oxygen consumption rate (OCR) was measured using the Seahorse XF24 extracellular flux analyser as previously described [33]. Briefly, cells were treated with DMSO or pyrvinium for 24 hours. Five replicate wells of 5×10^4^ drug-treated cells was seeded in 24-well XF24 well plates coated with BD Cell-Tak (BD Biosciences, MA, US) in unbuffered DMEM. Analyses were performed both at basal conditions and after injection of oligomycin (OLI, 1 μg/ml), Carbonyl cyanide-p-trifluoromethoxyphenylhydrazone (FCCP, 0.4 μM) and Antimycin A and Rotenone combination (A&R, 2.5 μM and 2.5 μM) according to the manufacturer's instructions. For time course analysis, OCR was measured using untreated cells both at basal conditions and after injection of various concentrations of pyrvinium. ATP levels were measured by CellTiter-Glo Luminiescent Cell Viability Assay (Promega, WI, US) according to the manufacturer's instructions.

### CML xenografts in SCID mouse

SCID mice at 6-8 weeks old were purchased from InVivos Pte Ltd Singapore. All procedures were conducted according to the guidelines approved by the Institutional Animal Care and Use Committee. Briefly, K562 (1×10^6^) cells in phosphate buffered saline were implanted subcutaneously into the right flank of each SCID mouse. Treatment was started when tumor volume reached approximately 200 mm^3^. The mice were treated with intraperitoneal pyrvinium 0.5 mg/kg daily, oral dasatinib 1 mg/kg daily or a combination of both. The control group was treated with intraperitoneal 50%/50% DMSO/saline and oral 80 mM citric acid. Tumor length and width were measured daily and volumes were calculated as (length)^2^ x (width)/2.

Additional methods can be found in the Supplemental Information.

## SUPPLEMENTARY MATERIAL TABLES AND FIGURES


