# Supplementary material for: Pyrvinium selectively targets blast phase-chronic myeloid leukemia through inhibition of mitochondrial respiration
Source: Oncotarget. 2015 Sep 10;6(32):33769–80. doi: 10.18632/oncotarget.5615 (PMC4741801; doi:10.18632/oncotarget.5615)
Supplement: Supplementary file 1 [file oncotarget-06-33769-s001.pdf]

Pyrvinium selectively targets blast phase-chronic myeloid leukemia through inhibition of mitochondrial respiration

Supplementary Material

Lists of content

Supplemental Methods 2

Supplemental Table S1: BP-CML Patients' information 3

Supplemental Table S3: Response of patient-derived and normal CB-derived CD34+ cells to pyrvinium treatment (via ex vivo colony formation assay). 5

Supplemental Table S4: Response of patient-derived and normal CB-derived CD34+ cells to pyrvinium treatment (via ex vivo serial replating assay). 6

Supplemental Figure S1. Pyrvinium and dasatinib treatment has no overt toxicity in mice. 7

Supplemental Figure S2. Combination of pyrvinium and imatinib/nilotinib is superior in inducing apoptosis than single drug alone. 8

Supplemental Figure S3. The efficacy of CK1α knockdown and β-catenin overexpression in leukemia , HCT-116 and SUM159 cells. 9

Supplemental Figure S4. Pyrvinium preferentially localizes in mitochondria of BP-CML cells and rapidly inhibiting mitochondrial respiratory capacity 10

Supplemental Figure S5. Pyrvinium decreases oxygen consumption in a rapid manner 11

Supplemental Figure S6. CML ρ^0 cells have reduced mitochondrial genomic content. 12

References 13

Supplemental Methods

Denaturing sodium dodecyl sulfate–polyacrylamide gel electrophoresis (SDS–PAGE) and Western blot (WB) analyses

Transfected cells were lysed by 4% SDS and total protein content was measured using the bicinchoninic acid protein assay kit (Thermo Scientific, NY, US). Proteins from whole cell extracts were resolved using denaturing SDS–PAGE and analyzed by WB. Antibodies used in WB analyses include anti-CK1α (C-19) (sc-6477, Santa Cruz, CA, US), and anti-PP2A A subunit (PP2A/A; JH242; in house) [1]. Immunoblots shown in the accompanying figures are the representative of three independent experiments.

Real-time RT-PCR

RNA was isolated from LAMA84, LAMA84 ρ^0, KU812 and KU812 ρ^0 cells with TRIzol Reagent (Life technologies, CA, US) and used to produce the first-strand cDNA with iScript cDNA Synthesis Kit (Bio-rad, CA). The cDNA was amplified via PCR using a SsoFast EvaGreen Supermix and CFX96 RT PCR system (Bio-rad, CA).The primers sets for human mitochondrially encoded NADH dehydrogenase 6 (*MT-ND6*) (5´-TAGGATTGGTGCTGTGGGTG-3' and 5´-TAATCATACAAAGCCCCCGC-3´), mitochondrially encoded cytochrome c oxidase II (*MT-CO2*) (5´-GATTGAAGCCCCCATTCGTA-3´and 5´- ACGATGGGCATGAAACTGTG -3') succinate dehydrogenase complex, subunit A, flavoprotein (Fp) (*SDHA*) (5´-AACTCGCTCTTGGACCTGGTTG-3´ and 5´-TCCGATGTTCTTATGCTTCCATCA-3´), and *β-actin* (5'-AAGGATTCCTATGTGGGCGACG-3' and 5'-GCCTGGATAGCAACGTACATGG-3'). The mRNA levels of *MT-ND6*, *MT-CO2*, and *SDHA* were quantified using a comparative CT method with *β-actin* levels for normalization.

Supplemental Table S1: BP-CML Patients' information

Patient Number	BCR-ABL1 mutation	BCR-ABL1 overexpression	TKI-resistant	Karyotype
BP-CML#1	None	NA	Newly diagnosed	46,XX,t(9;22)(q34;q11.2)[7]/46,idem,t(3;21)(q26.2;q22)[13]
BP-CML#2	E255K	No	IM, DAS, PON	48,XY,inv(3)(q21q26),+8,der(8)add(8)(p11.2)add(8)(q22),t(9;22)(q34;q11.2),+der(22)t(9;22)[20]
BP-CML#3	E453K	No	IM, NIL	Not available
BP-CML#4	None	No	DAS	46,XX,t(9;22)(q34;q11.2)[15]/46,idem,add(11)(p11.2)[4]/46,XX[1]
BP-CML#5	None	Yes	IM	45,XX,t(3;3)(q21;q26.1),-7,t(9;22)(q34;q11.2)[20]
BP-CML#6	None	No	IM	46,XY,inv(3)(q21q26.2),t(9;22)(q34;q11.2)[20]
BP-CML#7	E255V	No	IM	46,XY,t(7;9;22)(q36;q34;q11.2)[1]/46,idem,der(3)t(3;21)(q26.1;q11.2),add(21)(q11.2)[19]
BP-CML#8	None	Yes	IM, DAS	47~48,XX,der(5)t(1;5)(q11;q12),add(6)(q13),t(9;22)(q34;q11.2),add(15)(q11.2),+der(22)t(9;22)(q34;q11.2)[9]/46,XX,add(4)(p16),t(9;22)(q34;q11.2),del(11)(q13q23),add(16)(q12),add(17)(q12),del(20)(q11.2q13.3)[cp6]/46~47,XX,t(9;22)(q34;q11.2),der(13)t(1;13)(q21:q12)ins(13;?)(q12;?),+der(22)t(9;22)(q34;q11.2)[cp2]/46~47,XX,der(2)t(1;2)(q11;q13),add(4)(q31),add(6)(q23),t(9;22)(q34;q11.2),+der(22)t(9;22)(q34;q11.2)[cp2]/46,XX[1]

Notes: IM, imatinib; DAS, dasatinib; NIL, nilotinib; PON, ponatinib

Supplemental Table S2: Response of patient-derived and normal CB-derived CD34+ cells to pyrvinium treatment (via ex vivo apoptosis assay).

Apoptosis (% relative to control)										
	BP-CML #2	BP-CML #3	BP-CML #4	BP-CML #5	BP-CML #8	CB #1	CB #2	CB #3	CB #4	CB #5
Pyrvinium	2	3	2	1	0	1	2	2	3	1
Pyrvinium 100 nM	28	5	8	6	5	1	3	3	5	5
Pyrvinium 300 nM	32	25	32	36	26	1	10	5	6	7
Dasatinib 100 nM	6	7	13	38	10	2	7	7	3	6
Pyrvinium 300 nM + Dasatinib	58	49	59	71	85	2	23	12	13	14

Notes: Apoptosis assay using different CML and CB samples were performed under the same experimental conditions. Results shown are the percentage of induced apoptosis above the respective controls.

Supplemental Table S3: Response of patient-derived and normal CB-derived CD34+ cells to pyrvinium treatment (via ex vivo colony formation assay).

CFU (% relative to control)										
	BP-CML #1	BP-CML #3	BP-CML #5	BP-CML #6	BP-CML #7	CB #1	CB #2	CB #3	CB #4	CB #5
Pyrvinium 30 nM	87	99	66	80	83	100	100	101	94	98
Pyrvinium 100 nM	47	56	36	57	64	100	90	87	76	82
Dasatinib 100 nM	21	8	14	11	10	74	57	56	71	41
Pyrvinium 30 nM + Dasatinib	2	2	4	4	3	71	55	57	70	41

Notes: CFU assay using different CML and CB samples were performed under the same experimental conditions. Results shown are the percentage of number of colonies in the pyrvinium-treated relative to number of colonies in control.

Supplemental Table S4: Response of patient-derived and normal CB-derived CD34+ cells to pyrvinium treatment (via ex vivo serial replating assay).

% Serial replating efficiency										
	BP-CML #1	BP-CML #3	BP-CML #5	BP-CML #6	BP-CML #7	CB #1	CB #2	CB #3	CB #4	CB #5
Pyrvinium 0 nM	38	32	33	40	33	51	52	49	46	44
Pyrvinium 30 nM	28	27	26	33	28	51	50	49	45	38
Pyrvinium 100 nM	10	17	13	14	17	35	31	36	38	29
Dasatinib 100 nM	5	4	16	11	6	43	49	43	41	29
Pyrvinium 30 nM + Dasatinib	ND	ND	ND	ND	ND	44	47	44	41	27

Notes: Serial replating assay using different CML and CB samples were performed under the same experimental conditions. Results shown are the percentage of the final number of colonies among total number of colonies plated. ND, no colonies detected.

Figure S1

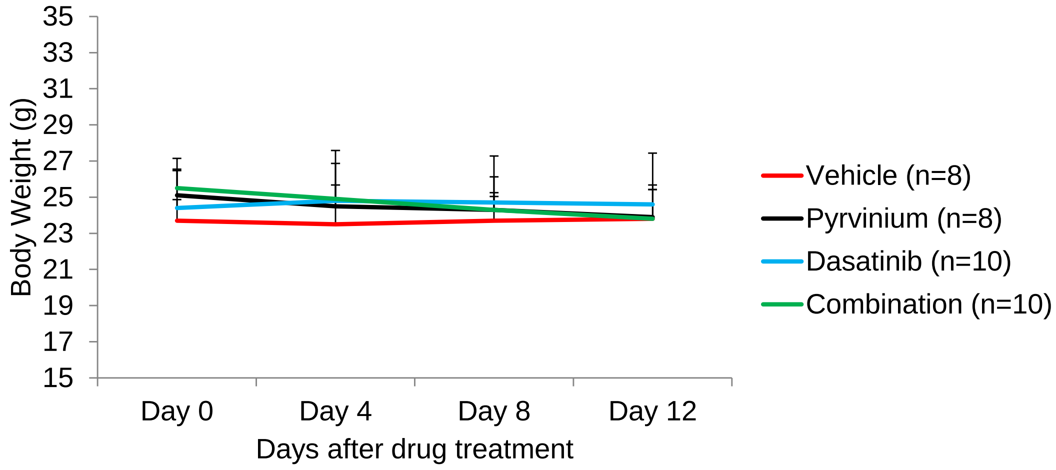

Supplemental Figure S1. Pyrvinium and dasatinib treatment has no overt toxicity in mice. The difference in mouse body weight across treatment groups is insignificant. Body weight of mice was measured every 3 days and mice were sacrificed when tumor volume reached 1500 mm^3.

Figure S2

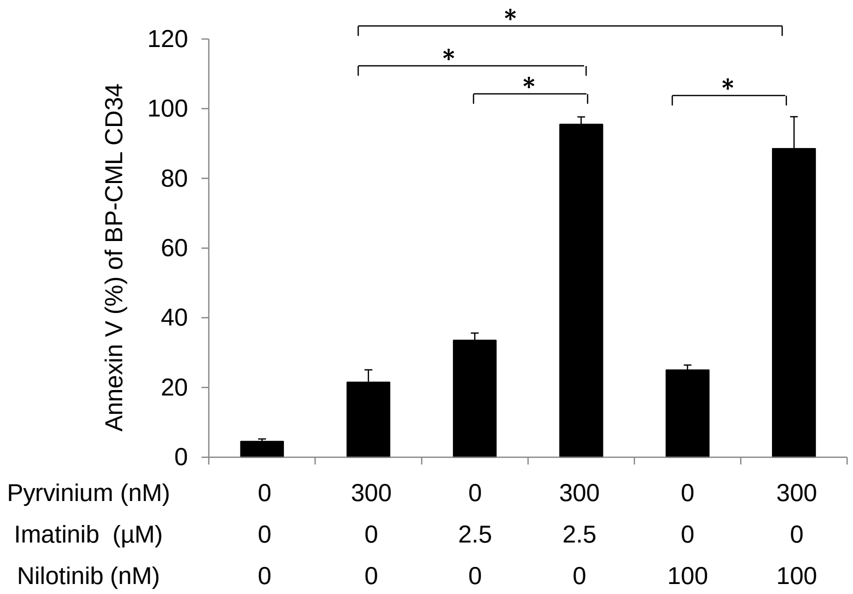

Supplemental Figure S2. Combination of pyrvinium and imatinib/nilotinib is superior in inducing apoptosis than single drug alone. Results shown are the average percentage of induced apoptosis obtained from two patient-derived BP-CML samples. *p<0.01, compared to single arm treatment.

Figure S3

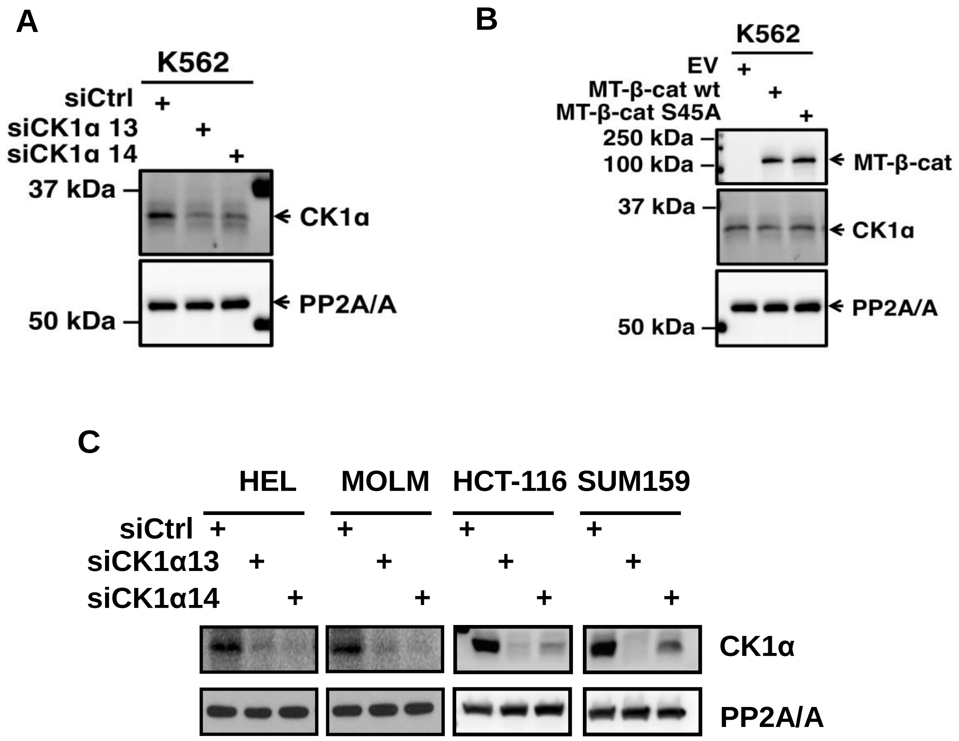

Supplemental Figure S3. The efficacy of CK1α knockdown and β-catenin overexpression in leukemia , HCT-116 and SUM159 cells. (a) Depletion of CK1α by 2 independent siRNAs in K562 CML cells. Cells are electroporated with 100 nM siCtrl or siCK1α (#13 or #14) and cultured for 24 hours prior to analysis. (b) Overexpression of Myc-tagged-β-catenin (MT-β-cat) variants in K562 CML cells. Cells are electroporated with 1.5 µg pCS2-MT (EV), pCS2-MT-β-cat wt or pCS2-MT-β-cat S45A and cultured for 24 hours prior to analysis. Anti-Myc antibody (9E10) was used in immunoblots to confirm overexpression of MT-β-cat variants in K562 cells (c) Depletion of CK1α in two AML cell lines HEL and MOLM, HCT-116 colon and SUM159 breast carcinoma cells by 2 independent siRNAs in K562 CML cells. Cells are electroporated with 100 nM siCtrl or siCK1α (#13 or #14) and cultured for 24 hours prior to analysis. PP2A A subunit (PP2A/A) serve as the loading control in immunoblots.

Figure S4

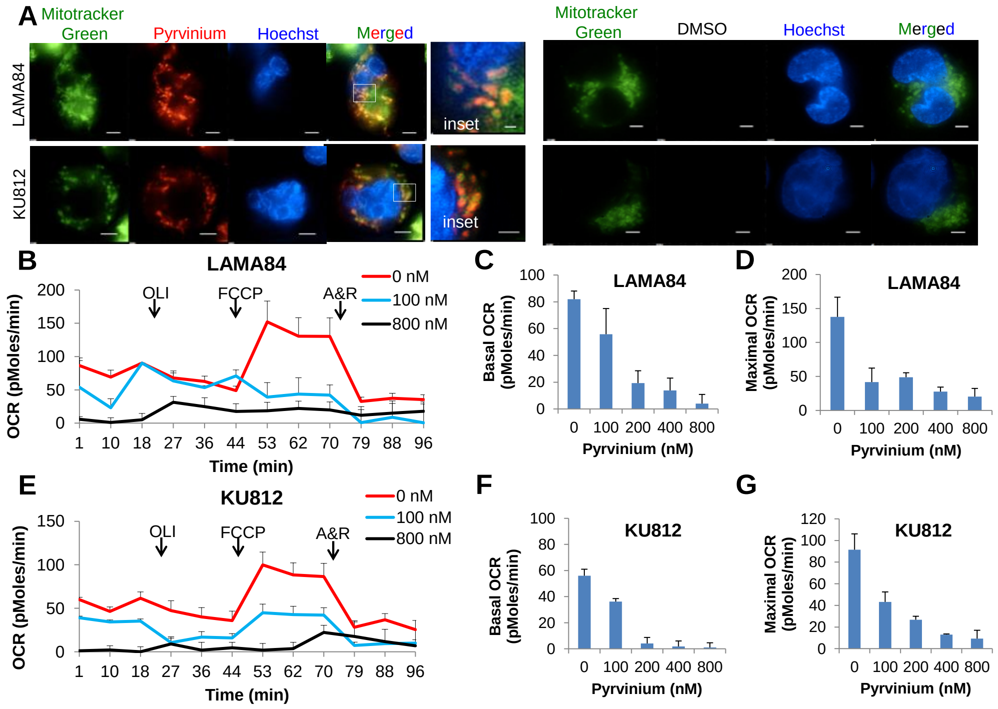

Supplemental Figure S4. Pyrvinium preferentially localizes in mitochondria of BP-CML cells and rapidly inhibiting mitochondrial respiratory capacity. (a) Pyrvinium preferentially localizes to the mitochondria of CML cells. Super resolution microscopy of LAMA84 and KU812 cells incubated with 200 nM pyrvinium (red) for 5 minutes, washed with plain RPMI-1640 and then incubated with Mitotracker to stain for mitochondria (green). Hoechst 33342 stains the nuclei. Scale bar is 5µm. (b – g). Pyrvinium significantly decreases basal and maximal OCR (oxygen consumption rate) in CML cells. LAMA84 and KU812 cells were treated with DMSO or pyrvinium for 24 hours and OCR was measured without (the first three measurements) and in the presence of (measurements 4-12) the mitochondrial inhibitors oligomycin (OLI, 1 µg/ml), FCCP (0.4 µM) and Antimycin A and Rotenone combination (A&R, 2.5 µM and 2.5 µM). Basal OCR is calculated as the mean of measurements of the first three. Maximal OCR is calculated as the mean of the measurement 7 – 9.

Figure S5

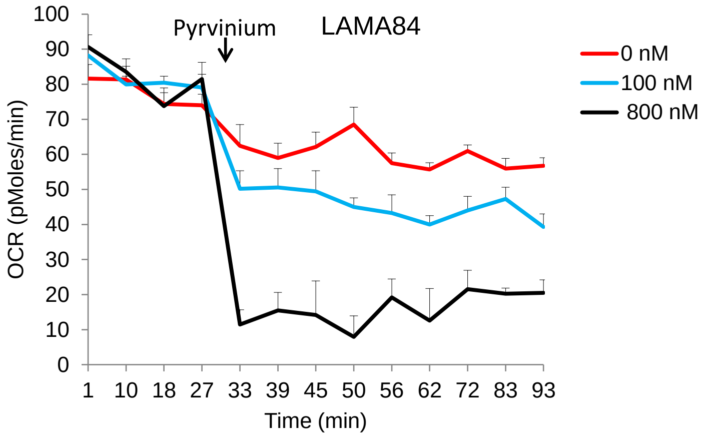

Supplemental Figure S5. Pyrvinium decreases oxygen consumption in a rapid manner. OCR was measured without (the first four measurements) and with different concentrations of pyrvinium (measurements 5-13) every 5 minutes.

Figure S6

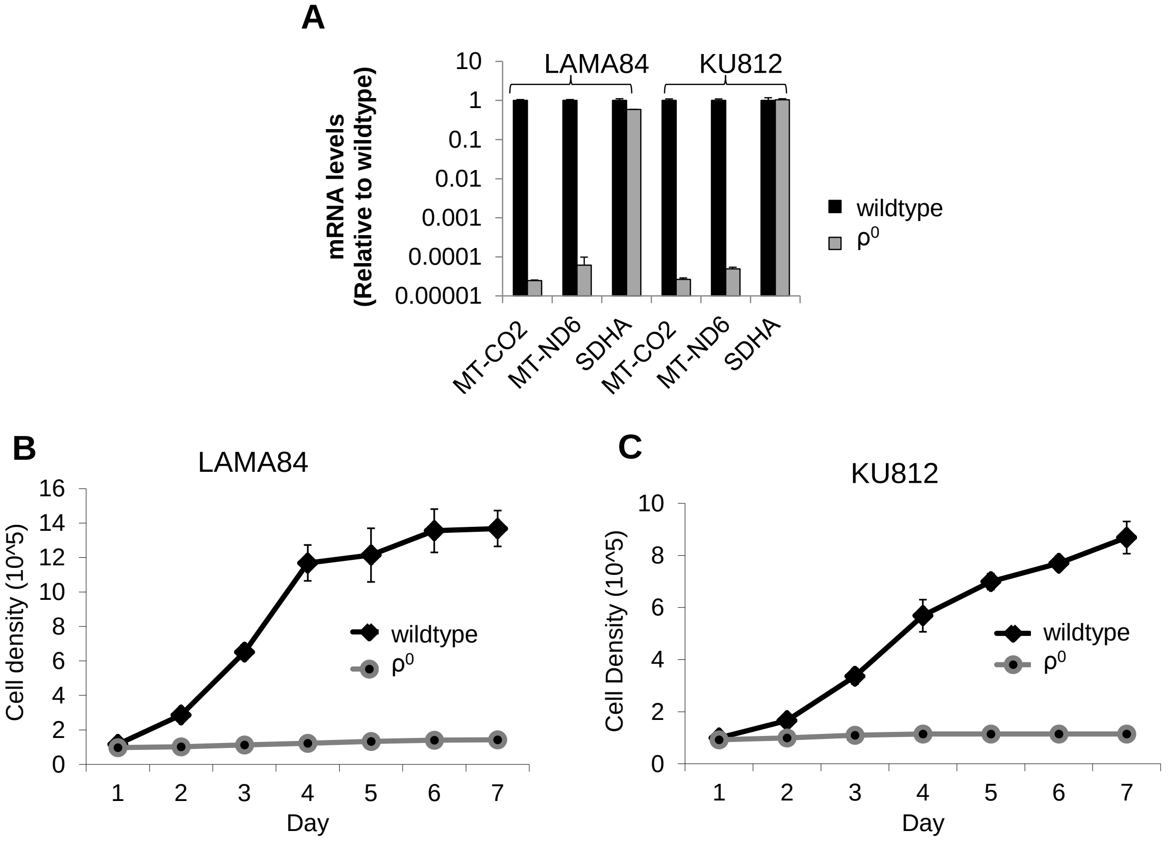

Supplemental Figure S6. CML ρ^0 cells have reduced mitochondrial genomic content. (A) Real-time RT-PCR expression profiling of two mitochondrial genome-encoded (*MT-ND6* and *MT-CO2*) and one nuclear genome-encoded (*SDHA*) respiratory chain enzyme subunits in LAMA84 and KU812 ρ^0 cells. Fold-expression change of the transcript abundance in CML ρ^0 is first normalized to *β-actin* expression and then calculated relative to its wildtype counterparts. MT-ND6 and MT-CO2 transcripts are 10^5 lower whereas SDHA is not affected in the ρ^0 CML cells. Proliferation of LAMA84 ρ^0 (B) and KU812 ρ^0 (C) is very minimal. Equal number of cells was seeded on day 1 and cell density was monitored every day for 6 days.

References

1. B. McCright and D. M. Virshup. Identification of a new family of protein phosphatase 2A regulatory subunits. J Biol Chem. 1995; 270: 26123-8.
